# Concurrent versus sequential immunotherapy with chemoradiotherapy for unresectable stage III non-small-cell lung cancer: a retrospective study

**DOI:** 10.3389/fonc.2024.1515382

**Published:** 2025-01-07

**Authors:** Zhou Meng-Xi, Fan Wen-Jie, Zhang Ning, Zhu Li-Yang, Wang Hong-Yan

**Affiliations:** Department of Radiation Oncology, The First Affiliated Hospital of Anhui Medical University, Hefei, Anhui, China

**Keywords:** immunotherapy, sequential immunotherapy, chemoradiotherapy, NSCLC, efficacy, safety

## Abstract

**Background:**

Immunotherapy combined with chemoradiotherapy has demonstrated promising efficacy in stage III non-small-cell lung cancer (NSCLC). However, the optimal timing for immunotherapy intervention during radiotherapy remains unclear. This study aimed to compare the efficacy and safety of immune checkpoint inhibitors (ICIs) administered concurrently or sequentially with chemoradiotherapy in unresectable stage III NSCLC.

**Methods:**

A retrospective analysis of 98 patients with unresectable stage III NSCLC, treated between January 1, 2019, and June 30, 2023, was conducted. Patients were grouped based on concurrent or sequential administration of ICIs with chemoradiotherapy. Median progression-free survival (mPFS), median overall survival (mOS), 1 and 2-year PFS rates, 2 and 3-year OS rates, objective remission rate (ORR), and disease control rate (DCR) were evaluated. Survival analysis was performed using the Kaplan–Meier method. Univariate and multivariate analyses were conducted using the log-rank test and Cox proportional hazards model. Treatment-related adverse effects were assessed and graded.

**Results:**

A total of 98 patients with unresectable stage III NSCLC treated with chemoradiotherapy and ICIs were included. The mPFS and mOS were 19.0 (14.2-23.8) months and 31.5 (24.3-38.7) months, 12.8 (9.5-16.1) months and 28.5 (19.3-37.7) months in the concurrent and sequential ICI groups, respectively, and mPFS showed a significant difference (P=0.047). The estimated 1 and 2-year PFS rates were 79.6% (95% confidence interval [CI]: 67.6-91.6) and 40.4% (95% CI: 15.8-49.2) for the concurrent group, compared to 51.0% (95% CI: 35.9-66.1) and 31.6% (95% CI: 14.5-48.7) for the sequential group. The estimated 2 and 3-year OS rates were 65.7% (95% confidence interval [CI]: 48.6-82.8) and 40.0% (95% CI: 16.1-63.9) for the concurrent group, compared to 54.6% (95% CI: 35.8-73.4) and 28.7% (95% CI: 4.8-52.6) for the sequential group. The Eastern Cooperative Oncology Group Performance Status Scale (ECOG) score and tumor differentiation were identified as independent factors associated with PFS and OS. Distant metastasis occurred in 13.8% and 25.5% of patients in the concurrent and sequential ICI groups, respectively (P=0.049). The incidence of any grade of pneumonitis was 43.1% and 38.3% in two groups, with grade 3 or higher in 7.8% and 8.5% of patients, respectively. Hematologic toxicity of any grade was observed in 29.4% and 34.0% of the two groups, with grade 3 or higher toxicity identified in 3.9% and 2.1% of patients, respectively.

**Conclusions:**

Concurrent immunotherapy combined with chemoradiotherapy demonstrated superior efficacy than sequential immunotherapy, with good safety and tolerability in patients with unresectable stage III NSCLC.

## Background

Non-small-cell lung cancer (NSCLC) is recognized for its high prevalence and malignancy (1, 2). Approximately one-third of patients with NSCLC are initially diagnosed at stage III (locally advanced) (3), for which the 2 and 5-year OS rates are less than 50% and 30%, respectively, highlighting the need for further therapeutic advancements (4). Stage III NSCLC is highly heterogeneous, for its variations in tumor size, adjacent invasion, and lymph node metastasis, contributing to the complexity and variability of treatment strategies (5). For unresectable stage III NSCLC, the current standard treatment for these cases is concurrent chemoradiotherapy (c-CRT). Although several multicenter studies have explored sequential surgery following c-CRT, the outcomes were suboptimal (6, 7). With the advent of immunotherapy, integrating c-CRT with immune checkpoint inhibitors (ICIs) targeting anti-programmed cell death-protein 1 (PD-1) or programmed death-ligand 1 (PD-L1) has become a new direction (8).

Currently, most trials and guidelines recommend c-CRT followed by sequential PD1/PD-L1 inhibitors. However, as many patients experience local recurrence or distant metastasis, the potential benefits of synchronizing immunotherapy with radiotherapy merit further exploration. Radiotherapy can enhance immune system activation, and in turn, radiation may amplify immunotherapy effects (9). Concurrent administration of immunotherapy and radiotherapy produces a synergistic effect, improving treatment response and OS compared to CRT with consolidation immunotherapy, regardless of PD-L1 expression levels (10). NICOLAS single-arm Phase II trial combined nivolumab concurrently with CRT followed by 12-month consolidation therapy. The trial reported preliminary promising outcomes (11). Therefore, combining CRT with PD1/PD-L1 inhibitors is a promising and rational strategy in cancer research, and its therapeutic efficacy and safety are still worthy of further exploration.

This retrospective study included 98 patients with unresectable stage III NSCLC who received either concurrent or sequential PD1/PD-L1 inhibitors with CRT between January 2019 and June 2023. The primary objectives were to evaluate clinical efficacy and monitor treatment-related adverse events (TRAEs), providing clinical data on the optimal timing for immunotherapy intervention in unresectable stage III NSCLC.

## Methods

### Population

A retrospective analysis was conducted on patients treated at the Department of Radiotherapy, the First Affiliated Hospital of Anhui Medical University, between January 2019 and June 2023. Patients were assigned to one of the two groups according to different treatment methods: (1) concurrent ICIs group, where PD1/PD-L1 inhibitors were administered during CRT (either within 2 weeks before the first radiation session or 2 weeks after the last session). (2) sequential ICIs group, where PD1/PD-L1 inhibitors were administered more than 2 weeks after completing radiotherapy.

Baseline demographic data, pathological diagnoses, imaging findings, laboratory tests, and treatment details were obtained from the hospital's medical record system. Disease staging followed the eighth edition of the TNM classification for NSCLC, established by the Union for International Cancer Control (UICC) and the American Joint Committee on Cancer (AJCC).

The inclusion criteria were: (1) Histologically confirmed NSCLC via fiberoptic bronchoscopy or percutaneous lung biopsy, including squamous cell carcinoma, adenocarcinoma. (2) Imaging-confirmed, locally advanced stage IIIA-C NSCLC (T1-2N2-3, T3N1-3, T4N0-3). (3) Inoperability. (4) Age ≥18 and ≤85 years. (5) Normal hemotology examination. (6) The Eastern Cooperative Oncology Group (ECOG) Performance Status Scale ≤2 points.

The exclusion criteria included: (1) Presence of other primary tumors. (2) History of lung cancer surgery or radiotherapy. (3) Autoimmune diseases. (4) History of PD-1/PD-L1 inhibitors. (5) Active hepatitis. (6) Mutated driver genes. (7) Mixed small cell histological features. (8) Disease progression post-radiotherapy.

### Procedures

All patients received intensity-modulated radiation therapy (IMRT). Gross tumor volume (GTV) encompassed the primary lung tumor and any mediastinal or hilar lymph nodes with confirmed metastasis, as identified by thoracic computed tomography (CT) examination or positron emission tomography-CT (PET-CT). Clinical target volume (CTV) was generated by expanding the GTV by a 6-8 mm margin, covering portions of the hilar and mediastinal lymphatic drainage areas. The CTV was minimized when in proximity to critical organs. The planning target volume (PTV) was expanded by a 3 mm margin from the CTV. A prescribed dose of 56 to 66 Gy was delivered to 95%PTV over 28 to 33 once-daily fractions (56-66 Gy/28-33 f), administered five days a week, Monday to Friday.

All patients underwent chemotherapy concurrently and following radiotherapy. Chemotherapeutic agents included cisplatin/carboplatin, paclitaxel liposome/albumin-bound paclitaxel/docetaxel, or pemetrexed disodium, administered every 3 to 4 weeks for 4 to 6 cycles. In the sequential ICIs group, PD1/PD-L1 inhibitors were initiated more than 2 weeks after completing CRT. In the concurrent ICIs group, PD1/PD-L1 inhibitors were administered during CRT or within 2 weeks before or after radiotherapy; the inhibitors used included envafolimab, sugemalimab, pembrolizumab, camrelizumab, sintilimab, and tislelizumab, all of which are approved for NSCLC treatment. These inhibitors were given every 3 to 4 weeks until disease progressed, death. or intolerable side effects, with a maximum treatment period of 12 months.

Before each treatment cycle, patients underwent routine blood tests (routine blood examination, liver and kidney function, thyroid hormone levels, myocardio enzyme, lung cancer tumor markers). Enhanced thoracic and abdominal CT and cardiac ultrasounds were conducted every two treatment cycles.

### Outcomes

The primary endpoints were PFS, OS, 1/2-year PFS, and 2/3-year OS rates. PFS was from the start of the radiotherapy to tumor progression, while OS was from the start of radiotherapy to death. The secondary endpoints were as follows: complete response (CR), partial response (PR), stable disease (SD), and progressive disease (PD). Disease progression was classified into locoregional progression and distant metastasis. The ORR was calculated as (CR+PR)/total number of cases×100%, while the disease control rate (DCR) was defined as (CR+PR+SD)/total number of cases×100%. The therapeutic response was assessed by investigators according to Response Evaluation Criteria In Solid Tumors (RECIST 1.1) using thoracic and abdominal CT examination. The first time of CT examination was one month after the radiotherapy. Besides, TRAEs were closely monitored, focusing on pneumonitis and hematological toxicity. Other common TRAEs, including radiation esophagitis, liver dysfunction, hypothyroidism, asthenia, decreased appetite, and diarrhea, were also documented.

### Follow-up

Acute toxicities occurring within 3 months post-radiotherapy were observed and graded. Thoracic and abdominal CT examinations were performed every 2-3 months within 2 years and 6 months after 2 years for response evaluation. Bone emission computed tomography (ECT) and brain magnetic resonance imaging (MRI) were performed annually. Blood test, including routine blood examination, liver and kidney function, tumor markers were tested at the same time as imaging examination. These examinations could be performed at any time when new or severe clinical symptoms appeared. The final follow-up was completed on June 30, 2023.

### Statistical analysis

Data analysis was performed using Statistical Package for the Social Sciences (SPSS; version 26.0, International Business Machines Corporation, New York, USA) and GraphPad Prism 7.0 (GraphPad Software, Inc., San Diego, California). Categorical data was expressed as percentages (%). Comparisons between groups were made using the chi-square or Fisher’s exact test. Kaplan–Meier method was used for survival analysis and survival curves, while univariate analysis was conducted using the log-rank test. Multivariate analysis for prognostic factors was performed using the Cox proportional hazards model. Statistical significance was set at P<0.05.

## Results

### Baseline characteristics

98 eligible patients with stage III NSCLC were included in the study. They were assigned to concurrent or sequential immunotherapy groups based on their treatment regimens. No significant differences were observed between the groups in terms of age, sex, ECOG scores, histological classification and grade, tumor location, previous treatments, or smoking and drinking history. The baseline characteristics of the patients are shown in **Table 1**.

**Table 1:** Baseline characteristics of patients n(%).

Characteristics	Concurrent ICIs (n = 51)	Sequential ICIs (n = 47)	*x^2^*	*p*
Gender				
Male	46 (90.2)	36 (76.6)	3.312	0.069
Female	5 (9.8)	11 (23.4)		
Age, years				
≥65	26 (51.0)	30 (63.8)	1.649	0.199
<65	25 (49.0)	17 (36.2)		
ECOG				
0	18 (35.3)	15 (31.9)	0.127	0.939
1	30 (58.8)	29 (61.7)		
2	3 (5.9)	3 (6.4)		
Histological classification				
Squamous	32 (62.8)	28 (59.6)	0.104	0.748
Adenocarcinoma	19 (37.3)	19 (40.4)		
Differentiation				
Poorly differentiated	18 (35.3)	16 (34.0)	0.017	0.897
Moderate+well differentiated	33 (64.7)	31 (66.0)		
Location				
Center lung	26 (51.0)	18 (38.3)	1.590	0.207
Right lung	25 (49.0)	29 (61.7)		
Diagnosdic method				
Fiberoptic bronchoscopy	25 (49.0)	21 (44.7)	0.539	0.764
Percutaneous lung puncture	24 (47.1)	25 (53.2)		
Lymph node puncture	2 (3.9)	1 (2.1)		
History of smoking				
Yes	23 (45.1)	21 (44.68)	0.002	0.967
No	28 (54.9)	26 (55.32)		
History of drinking				
Yes	16 (31.4)	9 (19.2)	1.923	0.166
No	35 (68.6)	38 (80.9)		

### Response evaluation and efficacy

As of the last follow-up on June 30, 2023, neither group achieved CR of their targeted tumors. In the concurrent ICIs group, 15 patients (29.4%) achieved PR, 10 (19.6%) had SD, and 26 (51.0%) experienced PD. In the sequential ICIs group, ten patients (23.4%) achieved PR, 7 (14.9%) had SD, and 30 (61.7%) experienced PD. The ORR and DCR were higher in the concurrent ICIs group (29.4% vs. 23.4% for ORR) and (49.0% vs. 38.3% for DCR) (**Table 2**).

**Table 2:** Response evaluation and efficacy of treatment one month after radiotherapy n(%).

Response evaluation	Concurrent ICIs (n = 51) (n=53)	Sequential ICIs (n = 47)	*p*
CR	0 (0.0)	0 (0.0)	
PR	15 (29.4)	11 (23.4)	
SD	10 (19.6)	7 (14.9)	
PD	26 (51.0)	29 (61.7)	
ORR (%)	29.4	23.4	0.501
DCR (%)	49.0	38.3	0.285

### Survival analysis

**Figure 1:**
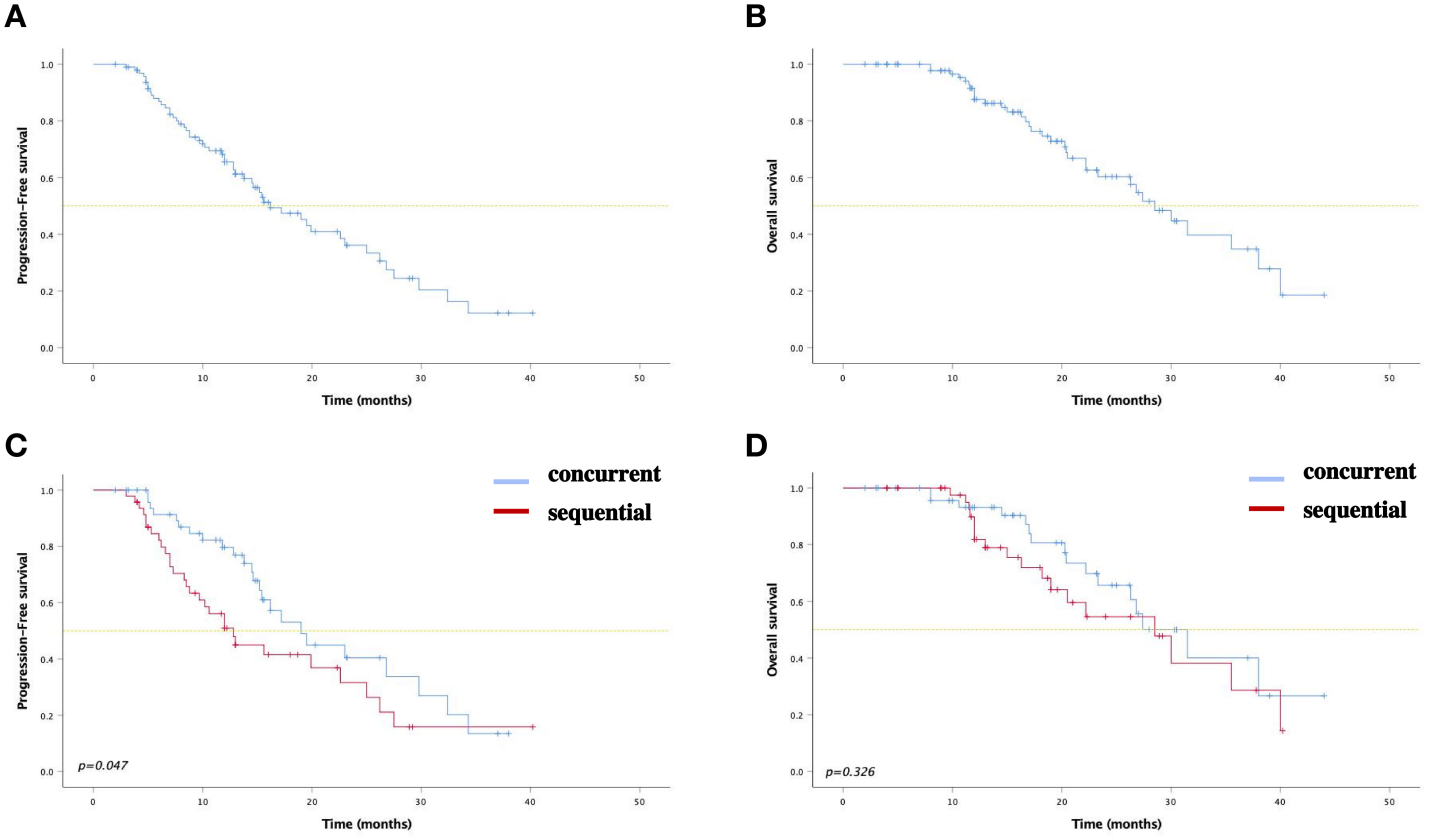
Kaplan–Meier curves for **(A)** PFS and **(B)** OS among all patients. Kaplan–Meier curves for **(C)** PFS and **(D)** OS between two groups.

At the last follow-up on June 30, 2023, the median PFS (mPFS) was 19.0 months (95% confidence interval [CI]: 14.2-23.8) and 12.8 months (95% CI: 9.5-16.1) for the concurrent and sequential ICI groups, respectively (P=0.048). The 1- and 2-year PFS rates were 79.6% (95% CI: 67.6-91.6) and 40.4% (95% CI: 22.2-58.6) in the concurrent ICI group, 51.0% (95% CI:35.9-66.1) and 31.6% (95% CI: 14.5-48.7) in the sequential ICI group. The median OS was 31.5 months (95% CI: 24.3-38.7) in the concurrent ICI group and 28.5 months (95% CI: 19.3-37.7) in the sequential ICI group (P=0.326). The 2- and 3-year OS rates were 65.7% (95% CI: 48.6-82.8) and 40.0% (95% CI: 16.1-63.9) in the concurrent ICI group, 54.6% (95% CI:35.8-73.4) and 28.7% (95% CI: 4.8-52.6) in the sequential ICI group, respectively. Survival curves and detailed survival analysis are presented in **Figure 1** and **Table 3**.

**Table 3:** Survival analysis of PFS and OS for patients.

Survival analysis	Concurrent ICIs	Sequential ICIs	x^2^	*p*
median PFS, month (95% CI)	19.0 (14.2-23.8)	12.8 (9.5-16.1)	3.939	0.047*
1-year PFS rate, % (95% CI)	79.6 (67.6-91.6)	51.0 (35.9-66.1)		
2-year PFS rate, % (95% CI)	40.4 (22.2-58.6)	31.6 (14.5-48.7)		
median OS, month (95% CI)	31.5 (24.3-38.7)	28.5 (19.3-37.7)	0.965	0.326
2-year OS rate, % (95% CI)	65.7 (48.6-82.8)	54.6 (35.8-73.4)		
3-year OS rate, % (95% CI)	40.0 (16.1-63.9)	28.7 (4.8-52.6)		

* p<0.05.

### Cumulative incidence of locoregional progression and distant metastasis

**Figure 2:**
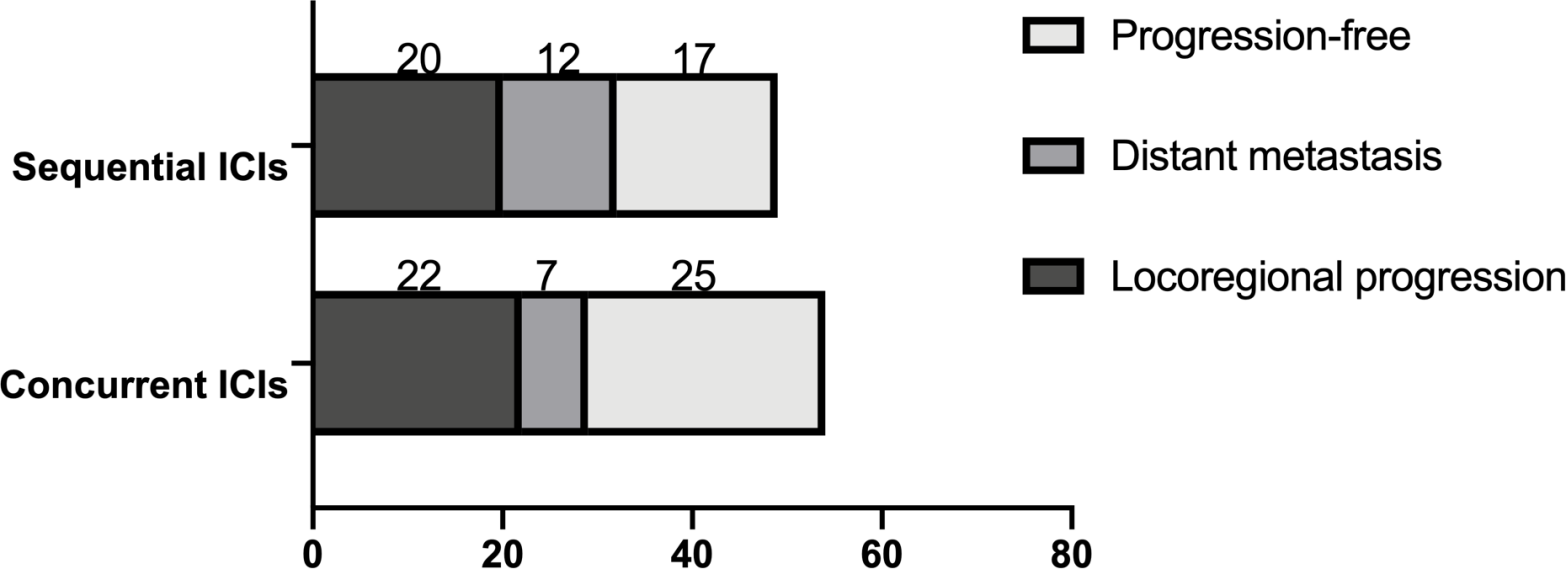
Patterns of disease progression, including locoregional progression and distant metastasis.

**Figure 3:**
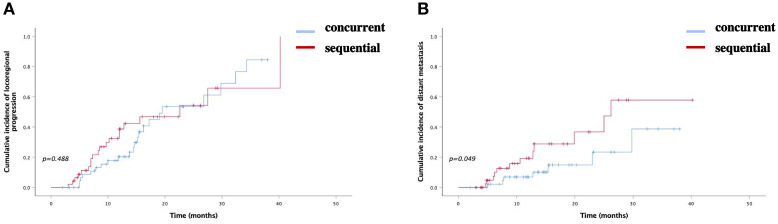
Cumulative incidence of **(A)** locoregional progression and **(B)** distant metastasis bewteen two groups.

In the concurrent ICI group, 22 patients (43.14%) experienced locoregional progression, compared to 20 (42.55%) in the sequential ICI groups (P=0.488). The 1-year and 2-year incidence rates of locoregional progression were 20.3% and 53.4% in the concurrent ICIs group, and 38.7% and 54.3% in the sequential ICI group. Distant metastasis occurred in seven patients (13.73%) in the concurrent ICIs group and twelve (25.53%) in the sequential ICI group, showing a significant difference (P=0.049). The 1-year and 2-year incidence rates were 10.2% and 23.4% in the concurrent ICI group, compared to 19.3% and 36.7% in the sequential ICI group (**Figures 2**, **3**).

### Univariate and multivariate analysis for PFS

Univariate analysis identified ECOG score, histological classification, and tumor differentiation as significant predictors of PFS and OS. Patients with moderate to well tumor differentiation (hazard ratio [HR]=0.300, 95% CI: 0.164-0.549, P<0.001), and (HR=0.267, 95% CI: 0.133-0.539, P=0.013), and those with adenocarcinoma (HR=0.471, 95% CI: 0.260-0.855, P<0.001) and (HR=0.348, 95% CI: 0.158-0.765, P=0.009) experienced significantly prolonged mPFS and mOS, respectively. Conversely, patients with an ECOG score of 2 (HR=2.619, 95% CI:1.424-4.818, P=0.002) and (HR=2.686, 95% CI:1.133-6.369, P=0.025) had a considerably shorter mPFS and mOS, respectively. These factors were further examined in multivariate Cox regression analysis, which confirmed that higher tumor differentiation and an ECOG score of 0-1 were independently associated with improved PFS and OS, with significant differences (**Tables 4**, **5**).

**Table 4:** Univariate and multivariate cox regression model of PFS for patients.

Characteristics	Univariate			Multivariate		
	HR	95% CI	*p*	HR	95% CI	*p*
Gender						
Male	1		0.314			
Female	1.409	0.720-2.756				
Age, years						
≥65	1		0.456			
<65	1.231	0.713-2.123				
ECOG						
0	1		0.002	1		0.011*
1-2	2.619	1.424-4.818		2.251	1.201-4.219	
Histological classification						
Squamous	1		0.013	1		0.179
Adenocarcinoma	0.471	0.260-0.855		0.657	0.356-1.212	
Differentiation						
Poorly	1		<0.001	1		0.001*
Moderate+well	0.300	0.164-0.549		0.330	0.175-0.623	
Location						
Center lung	1		0.591			
Right lung	0.862	0.500-1.484				
History of smoking						
Yes	1		0.499			
No	0.829	0.482-1.427				
History of drinking						
Yes	1		0.687			
No	0.884	0.485-1.610				

* p<0.05.

**Table 5:** Univariate and multivariate cox regression model of OS for patients.

Characteristics	Univariate			Multivariate		
	HR	95% CI	*p*	HR	95% CI	*p*
Gender						
Male	1		0.811			
Female	0.897	0.367-2.179				
Age, years						
≥65	1		0.370			
<65	1.376	0.685-2.762				
ECOG						
0	1		0.022	1		0.047*
1-2	2.429	1.137-5.186		2.196	1.012-4.767	
Histological classification						
Squamous	1		0.008	1		0.053
Adenocarcinoma	0.323	0.140-0.747		0.431	0.183-1.013	
Differentiation						
Poorly	1		0.001	1		0.014*
Moderate+well	0.279	0.131-0.595		0.366	0.164-0.816	
Location						
Center lung	1		0.246			
Right lung	1.152	0.750-3.084				
History of smoking						
Yes	1		0.183			
No	0.625	0.313-1.247				
History of drinking						
Yes	1		0.725			
No	0.875	0.414-1.844				

* p<0.05.

### Treatment-related adverse events

The incidence and severity of TRAEs were observed, focusing on pneumonitis and hematological toxicity. The overall incidence of pneumonitis was 43.1% and 38.3% in the concurrent and sequential ICI group, with grade 3 or higher reported in 7.8% and 8.5% of patients, respectively. The incidence of hematological toxicity was 29.4% and 34.0%, with grade 3 or higher occurring in 3.9% and 2.1% in the concurrent and sequential ICI group, respectively. Incidences of other TRAEs, including radiation esophagitis (9.8% vs. 12.8%), myocarditis (2.0% vs. 2.1%), abnormal liver function (19.6% vs. 19.2%), hypothyroidism (31.4% vs. 25.5%), asthenia (29.4% vs. 27.7%), decreased appetite (45.1% vs. 44.7%), and diarrhea (2.0% vs. 4.3%), were comparable between two groups (**Table 6**). Most TRAEs were grade 1/2 and resolved with symptomatic treatment. No serious TRAEs led to treatment termination.

**Table 6:** Treated-related adverse events of patients n(%).

TRAEs	Concurrent ICIs (n = 51)	Sequential ICIs (n = 47)	*p*
Pneumonitis	22 (43.1)	18 (38.3)	0.626
Grade 3/4 pneumonitis	4 (7.8)	4 (8.5)	0.904
Hematological toxicity	15 (29.4)	16 (34.0)	0.622
Grade 3/4 hematological toxicity	2 (3.9)	1 (2.1)	0.607
Radiation esophagitis	5 (9.8)	6 (12.8)	0.751
Myocarditis	1 (2.0)	1 (2.1)	0.932
Abnormal liver function	10 (19.6)	9 (19.2)	0.972
Hypothyroidism	16 (31.4)	12 (25.5)	0.605
Asthenia	15 (29.4)	13 (27.7)	0.943
Decreased appetite	23 (45.1)	21 (44.7)	0.897
Diarrhoea	1 (2.0)	2 (4.3)	0.600

## Discussions

The PACIFIC trial reported higher 2-year OS (66.3% vs. 55.6%) and longer mPFS (17.2 vs. 5.6 months) in the durvalumab group in patients with stage III NSCLC. These findings suggest that adding durvalumab to radiotherapy significantly improves PFS and OS outcomes (12). Zhou et al. (13) initiated a study in which patients were treated with different dose of radiation depending on tumor volume, followed by sequential sintilimab. The study presented a promising result in ORR and mPFS (60.7% and 8.6 months), while mOS was not reached. These landmark studies confirm the superior efficacy of combining immunotherapy with c-CRT over c-CRT alone in NSCLC.

With the significant survival benefits observed with immunotherapy, current guidelines recommend c-CRT combined with immunotherapy as the standard treatment for unresectable stage III NSCLC. However, there is limited research on the optimal timing for initiating immunotherapy. Our study addressed this gap by comparing the effectiveness of introducing immunotherapy during versus after c-CRT. Our results demonstrated that concurrent administration of ICIs with c-CRT yielded better outcomes than administering ICIs after CRT. The concurrent ICIs group showed longer mPFS (19.0 vs. 12.8 months) and mOS (26.8 vs. 22.2 months), higher 1-year (79.6% vs. 51.0%) and 2-year PFS rates (40.0% vs. 31.6%), higher 2-year (65.7% vs. 54.6% ) and 3-year OS rates (40.0% vs. 28.7%) superior ORR (29.4% vs. 23.4%), and higher DCR (49.0% vs. 38.3%) compared to the sequential ICIs group. These results mirror findings from DETERRED trial, which divided patients into groups receiving CRT with or without atezolizumab. The findings demonstrated that combining atezolizumab with CRT prolonged mPFS (13.2 vs. 12.5 months) and mOS (not reached vs. 22.8 months) (14). Similarly, the DOLPHIN trial showed promising results for patients with locally advanced NSCLC. The concurrent use of durvalumab with radiotherapy, followed by durvalumab consolidation, resulted in an mPFS of 25.6 months, a 1-year PFS rate of 72.1%, as well as a confirmed ORR of 90.9%, all of which were encouraging (15). A subgroup analysis from the PACIFIC trial reported that administering durvalumab within 14 days of the last radiation session significantly reduced the risk of disease progression (HR: 0.39 vs. 0.63) and increased the ORR (34.2% vs. 26.5%) compared to treatment initiated later than 14 days. This observation suggests that earlier introduction of immunotherapy after c-CRT may provide more significant benefits (16). NICOLAS Phase II trial arranged patients received nivolumab concurrently with CRT as well as nivolumab maintenance for up to a year post-radiotherapy. The trial reported promising outcomes in mPFS and mOS (12.7 and 38.8 months), as well as in 12-month PFS and 24-month OS rate (53.7% and 63.7%) (11). However, some trials have presented conflicting results. Zhao et al. (17) retrospectively analyzed c-CRT with concurrent or consolidation immunotherapy, and presented similarity in mPFS (22.6 vs. 24.6 months), with the ORR being higher in the immunotherapy consolidation group (67.57% vs. 42.19%). These variations could be attributed to biases resulting from limited sample sizes. Most landmark trials, in line with our study, demonstrate that concurrent immunotherapy with c-CRT yields superior efficacy compared to immunotherapy administered as consolidation after c-CRT.

The underlying reasons for these findings may be attributed to several factors. First, cell surface markers decreased in various tumor cells, contributing significantly to immune resistance and immune evasion (18). Radiation therapy has been shown to upregulate tumor-associated antigens levels on tumor cell surface, thereby addressing immune resistance and escape (19). Second, radiation gradually disintegrates tumor cells, releasing large quantities of tumor-associated antigens into the systemic circulation (20). These antigens stimulate T lymphocytes’ activation, differentiation, and sensitivity, enhancing the immune system’s ability to respond swiftly to abnormal cells (21). Third, radiation induces immune cells to migrate to the irradiated tumor site, a process known as the homing effect. This enhanced homing of immune cells helps modulate the tumor microenvironment, creating favorable conditions for immune responses and aiding in eliminating residual tumor cells (22). Our previous results confirmed higher 1-year PFS rate in the concurrent ICIs group, while no marked difference was found in 2-year PFS rate. Similarly, higher 2-year OS rate was found in concurrent ICIs group, while 3-year OS rate was almost the same. This finding might be due to the time-limited mutual sensitization effect of radiotherapy and immunotherapy. Without the continued stimulation of radiation, the impact of immunotherapy may diminish due to the reduced availability of circulating tumor antigens and recruitment of immune cells. The optimal sequencing of radiotherapy and immunotherapy is still under investigation to achieve more durable and effective immune responses.

Survival analysis revealed similar cumulative incidences of locoregional progression between the concurrent and sequential ICI groups (43.14% vs. 42.55%, P=0.488). However, the simultaneous use of ICIs with c-CRT significantly reduced the incidence of distant metastasis (13.73% vs. 25.53%, P=0.049). The reduction is due to modulation of tumor and immune microenvironment by radiation, enhancing immune surveillance against micrometastatic tumor cells (3). Furthermore, multivariate analysis indicated that patients with moderately or well differentiated tumors and those with better ECOG scores (0-1) had longer mPFS, mOS and higher survival rates. The higher the degree of differentiation of tumor cells, the smaller the atypia and the lower the malignancy, so that it is not easy to for tumor cells to invade the surrounding normal tissues, or result in distant metastasis. The ECOG score indicates a patient's physical fitness and ability to tolerate medical treatments. Most patients with low ECOG scores can complete full-dose anti-tumor treatments (including radiotherapy and medical therapies) on schedule. Conversely, those with higher ECOG scores may require reduced treatment intensity or prolonged treatment cycles, potentially compromising therapeutic efficacy. Therefore, ECOG scores and tumor differentiation can serve as vital prognostic indicators for patients with tumors.

A primary concern regarding c-CRT combined with immunotherapy for NSCLC is the potential increase in pneumonitis (including radiation-induced and immune-mediated) and hematological toxicity. Our study showed that the overall occurrence rate of pneumonitis was slightly higher in concurrent ICI groups (43.1 vs 38.3%). The grade 3 or higher pneumonitis rates were similar, at 7.8% and 8.5%. Chang et al. (23) compared the incidence of pneumonitis following stereotactic ablative radiotherapy (SABR) alone versus SABR combined with PD1/PD-L1 inhibitors (I-SABR) in those with NSCLC. The incidence of grade 1-2 pneumonitis was rare, and no cases of higher grade were reported in either group. Another trial, which administered durvalumab within 42 days of the final radiotherapy dose in patients with unresectable stage III NSCLC, reported a 16.2% incidence of grade 1-2 pneumonitis, a relatively high rate. However, pneumonitis of higher grade was observed in only 2.6% of patients (24). In the DOLPHIN trial, 23 patients developed pneumonitis of varying grades, though only four cases were grade 3 or higher (15). A meta-analysis which included 38 studies proved that the incidence of pneumonitis of any grade and grade ≥3 was similar in concurrent and consolidation ICI groups. However, CRT after ICI showed higher incidence of pneumonitis, which needed further confirmation (25). These findings suggest that combining PD1/PD-L1 inhibitors with radiotherapy is a promising treatment option with manageable side effects. While mild to moderate pneumonitis may occur more frequently with concurrent or sequential CRT and immunotherapy, severe pneumonitis remains rare.

The other significant concern with TRAEs in our study was hematological toxicity. The total incidences of hematological toxicity in the concurrent and sequential ICI groups were 29.4% and 34.0%. The incidences of grade 3 or higher toxicity were 3.9% and 2.1%, respectively, showing comparable results. Lin et al. (14) reported that patients with unresectable NSCLC receiving concurrent atezolizumab and CRT did not experience higher rates of decreased white blood cells or neutrophils. Only one case of grade 4 neutropenia was attributed to c-CRT rather than immunotherapy. Other adverse events showed similar rates between the groups, including radiation esophagitis (9.80% vs. 12.77%), myocarditis (1.96% vs. 2.13%), abnormal liver function (19.61% vs. 19.15%), hypothyroidism (31,37% vs. 25.53%), asthenia (29.41% vs. 27.66%), decreased appetite (45.10% vs. 44.68%), and diarrhea (1.96% vs. 4.26%). The CLOVER study, which divided patients into three groups receiving different chemotherapy drugs combined with immunotherapy and radiotherapy, found that the most common high grade TRAEs were neutropenia (51.6%), leukopenia (20.3%), and anemia (17.2%), primarily due to chemotherapy. The study hypothesized that concurrent durvalumab with CRT provided high efficacy with manageable safety (26). In conclusion, combining immunotherapy with c-CRT will not significantly increase the risk of TRAEs, especially pneumonitis and hematological toxicity.

## Conclusions

Concurrent chemoradiotherapy with immunotherapy in unresectable stage III NSCLC demonstrated superior efficacy to sequential immunotherapy, with favorable tolerance and safety profiles.

## Data Availability

The original contributions presented in the study are included in the article/supplementary material. Further inquiries can be directed to the corresponding author.
